# Long-lasting endothelium-dependent relaxation of isolated arteries caused by an extract from the bark of* Combretum leprosum*

**DOI:** 10.1590/S1679-45082015AO3242

**Published:** 2015

**Authors:** Francisco das Chagas Alves, Paulo Marques da Silva Cavalcanti, Rita de Cassia Aleixo Tostes Passaglia, Gustavo Ballejo

**Affiliations:** 1 Faculdade de Medicina de Ribeirão Preto, Universidade de São Paulo, Ribeirão Preto, SP, Brazil.; 2 Universidade Federal do Piauí, Teresina, PI, Brazil.

**Keywords:** Combretum, Arteries/drug effects, Endothelium/drug effects, Nitric oxide/pharmacology, Polyphenols/pharmacology

## Abstract

**Objective:**

To describe and to characterize the relaxing effect of an extract of the bark of *Combretum leprosum* on isolated arterial rings from different animals.

**Methods:**

Rings (3 to 4mm) from rabbit, rat, or porcine arteries rings were suspended in an organ bath (Krebs, 37°C, 95%O_2_/5%CO_2_) to record isometric contractions. After the stabilization period (2 to 3 hours) contractions were induced by the addition of phenylephrine (0.1 to 0.3µM) or U46619 (10 to 100nM), and *Combretum leprosum* extract was added on the plateau of the contractions. Experiments were performed to determine the potency, duration, reversibility, and to get insights on the potential mechanism involved in extract-induced relaxations.

**Results:**

In all rings tested, *Combretumleprosum* extract (1.5μg/mL) was able to cause relaxations, which were strictly endothelium-dependent. In rabbit or rat thoracic aorta rings, the relaxations were reversed by vitamin B_12a_ or L-N^G^-nitroarginine. In porcine right coronary arteries and rabbit abdominal aorta, extract caused both L-N^G^-nitroarginine-sensitive and L-N^G^-nitroarginine-resistant relaxations. In rabbit thoracic aorta, the extract was relatively potent (EC_50_=0.20µg/mL) and caused relaxations; intriguingly the endothelium continued to produce relaxing factors for a long period after removing the extract. The magnitude of extract-induced relaxations was significantly reduced in the absence of extracellular Ca^2+^; in addition, the TRPs channels blocker ruthenium red (10µM) was able to revert extract-induced relaxations. Phytochemical analyses indicated that the extract was rich in polyphenol-like reacting substances.

**Conclusions:**

*Combretum leprosum* extract contains bioactive compounds capable of promoting Ca^2+^-dependent stimulation of endothelial cells which results in a prolonged production of relaxing factors.

## INTRODUCTION

*Combretum leprosum *Mart. (Combretaceae) is a shrub or small tree that grows in northeastern Brazil where it is known as *mufumbo, mofumbo, cipoaba *and *pente-de-macaco*. In folk medicine, extracts from different parts of the plant are being used for alleged expectorant, hemostatic, sedative, and aphrodisiac properties.^1,2^

In a bioactivity-based screening of the potential effects of the lyophilized hydroalcoholic extract of *Combretum leprosum* (ECL) bark on visceral and vascular smooth muscles, it was observed that the extract caused a potent endothelium-dependent relaxation (EDR) of rabbit thoracic aorta rings. Although some substances isolated from related species of the *Combretum* genus have been shown to cause EDR in rat aorta (mollic acid glucoside, isolated from *Combretum molle*) as well as methanolic fractions rich in flavonoids from *Combretum celastroides* and *Combretum racemosum,* ^3,4^ all previously reported effects of flower, leaf and/or root ECL did not include any cardiovascular bioactivity.

Endothelial cells exhibit remarkable heterogeneity in responsiveness to agents that induce EDR either of homologous arteries from different animals species or of arteries from different vascular beds from the same animal; *e.g* acetylcholine (ACh), but not bradykinin (BK), induces EDR in the rabbit and rat aorta;^5,6^ similarly, histamine causes EDR in rat aorta but not in rabbit aorta. Therefore, it is highly recommended when describing the EDR of a new drug or natural product to examine its effects in same artery from more than one animal species and in different arteries from the same animal.

## OBJECTIVE

To describe and interpret the results of experiments conceived to answer the following questions regarding the observed novel effect of extract of *Combretum leprosum*: (1) Which is the site of action, the potency, and the duration of the effect of the extract of *Combretum leprosum* in provoking relaxations of the rabbit thoracic aorta rings? (2) Is the extract of *Combretum leprosum* capable of causing endothelium-dependent relaxation in isolated arterial rings from other vessels of the rabbit or from vessels of rat, mice, guinea-pig and pig? (3) Which are the possible mechanisms involved in the extract of *Combretum leprosum*-induced relaxations of rabbit thoracic aorta rings?

## METHODS

### Plant material

The stem bark of *C. leprosum* was collected in the morning (10 to 11:00am) on July 15, 2005, at the Agrarian Science Center, *Universidade Federal do Piauí*, Teresina (PI), Brazil. A voucher specimen (number 10,557) was deposited in the Graziela Barroso Herbarium (TEPB, at the same institution). The plant material was dried in the shade at 40±1°C, and the stem bark powder (500g) was extracted (three times) with 1L of 70% ethanol, evaporated in a vacuum at 50°C and lyophilized to obtain a dry extract, which was stored under refrigeration (4°C) until further use. The extract was freshly diluted in distilled water for experiments.

### Isolated organ experiments

Thirty male and female New Zealand rabbits (2.5 to 3.5kg) and 20 Wistar rats (200 to 250g), 5 male guinea-pigs (350g), and 10 male mice (24 to 30g) were employed for all the experiments. The animals were obtained from the vivarium of the *Ribeirão Preto Campus *of the* Universidade de São Paulo*. Hearts from ten pigs were obtained from a local slaughterhouse; they were removed immediately after the death of the animal, washed with cold Krebs solution to remove most of the blood, and transported to the laboratory in sealed plastic bags in a cooler box containing crushed ice. Within 2 to 3 hours after heart removal from the animals, the right and/or left coronary arteries were dissected out for preparation of the arterial rings. All experimental protocols were in accordance with the ethical principles for animal experimentation recommended by the *Colégio Brasileirode Experimentação Animal* [Brazilian College of Animal Experimentation] and were approved by the Animal Experimentation Ethics Committee (number 084/2011) of *Faculdade de Medicina de Ribeirão Preto* of *Universidade de São Paulo*.

Rats, mice, guinea-pigs, and rabbits were euthanized under sodium pentobarbital anesthesia (50mg/kg i.p. for rats, mice, and guinea-pigs and/or by marginal ear vein injection for rabbits). The thoracic descending aorta (from all animals) as well as the abdominal aorta, the superior mesenteric artery, and the common carotid artery (from rabbits) were quickly removed and placed in Krebs solution (116mM of sodium chloride − NaCl; 4.5nM potassium chloride - KCl; 1.14mM of monobasic sodium phosphate − NaH_2_PO_4_; 1.16nM of magnesium chloride − MgCl_2_; 2.5nM of Calcium chloride − CaCl_2_; 25nM od Sodium bicarbonate − NaHCO_3_; and 11.1 of D-glucose) containing diclofenac (10µM) to inhibit synthesis of cyclooxygenase derived-products.^6^ After all adherent tissues were removed, the arteries were cut into rings (3 to 4mm) taking care to preserve the endothelium; when required, the intima of some rings was rubbed with a metal stick to destroy the endothelium. The rings were suspended between thin metal brackets and mounted under an initial tension of 2 to 4g in isolated organ chambers (volume: 5 to 10mL) containing Krebs solution at 37±1°C bubbling continuously with 5% of carbon dioxide (CO_2_) and 95% of oxygen (O_2_). The isometric tension of each ring was continuously recorded and stored using the data acquisition system Lab Chart software.

### Experimental protocols

To test the viability of the rings, submaximal concentrations of phenylephrine (PE; 0.1 to 1μmol/L) or U46619 (10 to 30nmol/L) were added twice at hourly intervals. During the contraction plateau induced by the second addition of PE (or U46619), the functional integrity of the endothelium was tested by addition of ACh (1μmol/L) or BK (0.1μmol/L, pig coronary artery). Those rings in which ACh (or BK) caused a decrease of at least 80% of PE (or U46619)-induced tension were considered as containing intact functional endothelium. One hour after determining endothelium functionality, PE (or U46619) was again added to contract the rings and at the plateau of the contraction, and a supramaximal concentration of ECL (1.5μg/mL) was added.

To determine whether nitric oxide (NO) synthesis and/or release were required for ECL-induced relaxations, L-N^G^-nitro-L-arginine (L-NNA, 300μmol/L) or hydroxocobalamin (B_12a_; 30 to 100μM) were added when the relaxation response induced by ECL attained a steady state. To determine whether ECL-induced relaxations required the presence of extracellular Ca^2^, the effect of ECL was tested in preparations incubated in Krebs solution containing nominally zero Ca^2^. To determine whether Ca^2^ influx into endothelial cells was involved in ECL-induced relaxations, the effect of the addition of ruthenium red (RR; 10μmol/L, a non-selective blocker of Ca^2^ permeable channels) when the relaxation response induced by ECL attained a steady state was also examined.

The potency of ECL was calculated from concentration-effect curves obtained in a non-cumulative manner using intact endothelium rings from rabbit thoracic aortas, *i.e.* by adding single concentrations of ECL (0.1, 0.3, and 1.0µg/mL) on the plateau of PE-induced contractions at 45 to 60 minutes intervals. All drugs and the extract solutions were added in the medium chamber through a pipette in a volume of 1 to 30µL.

### Measurements of polyphenols

To estimate the total phenolic OH groups, the Folin-Ciocalteu (FC) method was utilized.^7^ The ECL (0.5mg/mL) solutions were diluted in aqueous sodium hydroxide (NaOH; 1%) to produce solutions with following concentrations: 1.0, 2.5, 5.0, 7.5, 10, and 12.5µg/mL. To these solutions, 500µL of FC reagent were added followed by vigorous shaking for 2 minutes. Later, 500µL of sodium carbonate (Na_2_CO_3_; 75g/mL) were added and these solutions were incubated at room temperature or at 50°C, for 20 minutes. Incubations were ended by cooling the solutions in ice water bath for 5 minutes. The specific absorbance at 700nm was determined using an Enzyme Linked Immuno Sorbent Assay (ELISA) plate reader; determinations were performed in triplicate. Solutions of gallic acid (GA) and quercetin (dissolved in 10% aqueous methanol) were utilized as standard polyphenol compounds.

### Statistical analyses

The ECL-induced relaxations of arterial rings were expressed as percentage of reduction (% tension) of the tension developed by PE- or U46619-induced contractions; the reversion of these relaxations by inhibitors was expressed as percentage of the relaxation magnitude (% relaxation). Values are presented as mean ± standard error of the mean (SEM). The ECL concentration causing half-maximal relaxation (EC_50_) was determined by fitting the original concentration-response curve to a sigmoidal curve using GraphPad Prism^®^ Software, version 5.0. Values (in percentage) of changes in the tonus were analyzed by the *t *Student test (two-tailed), paired (in the same arterial ring) or unpaired (in the different arterial rings). Values of p<0.05 were considered statistically significant.

### Chemicals

ACh, BK, FC reagent, GA, B_12a_, PE, quercetin, reagent grade ethanol, RR, and U46619 were obtained from Sigma (United States). Diclofenac was obtained from Calbiochem™ (United States). L-NNA was obtained from Research Biochemicals International (United States).

## RESULTS

**Figure 1:**
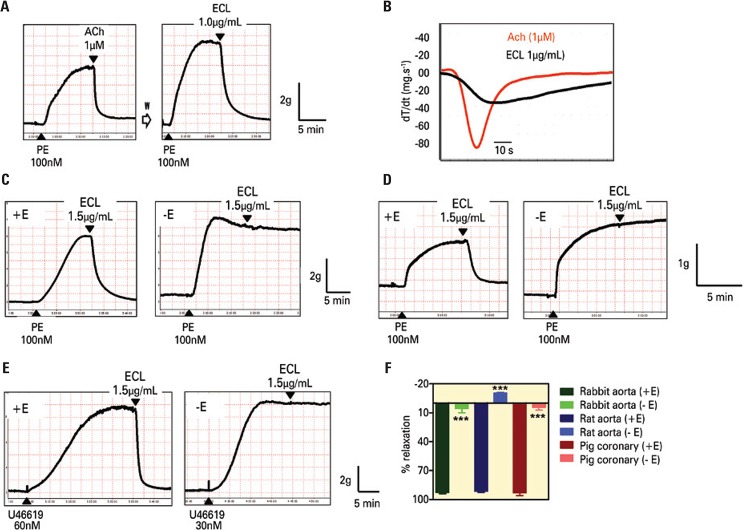
Representative tracings showing the effect of acetylcholine (ACh; 1µM) and extract of *Combretum leprosum* (ECL; 1.0µg/mL) on the isometric tension developed by rings of rabbit thoracic aorta pre-constricted with Phenylephrine (PE; 100nM) (A). The first derivative (dT/ dt, mg/s) of relaxing effects induced by ECL (1.0µg/mL) and ACh (1µM) (B). The effect of ECL (1.5µg/mL) on the isometric tension developed by rings, with (+E) or without (-E) endothelium, of rabbit thoracic aorta (C), rat thoracic aorta (D) pre-constricted with phenylephrine (PE), and porcine right coronary artery pre-constricted with U46619 (E). In (F), mean ± standard error of the mean (SEM) values of the magnitude of the relaxations observed in 4-20 similar experiments (relaxation magnitude is expressed as a percentage of reduction of PE- or U46619-induced contraction). ***Two-tailed p<0.0001 (unpaired t-test). W: washout of the preparation for 60-45 minutes

Initial experiments using rings from rabbit thoracic aorta with intact endothelium showed that an aqueous suspension of ECL at the concentration of 1.0 to 1.5µg/mL caused relaxations of magnitude similar to or even greater than those caused by ACh (1µM) (Figure 1A). While relaxations elicited by ACh were initiated almost immediately after its addition, those elicited by ECL required between 60 to 90 seconds to become apparent; furthermore while the relaxing effect of ACh reached a plateau in about 2 minutes, ECL induced effects took about 10 minutes to reach the plateau. The maximal velocity of relaxations, measured from the first derivative equation (dT/dt) were -90.33±5.41mg/s (n=8) and -39.83±2.28mg/s (n=8) for ACh (1µM) and ECL (1.0µg/mL), respectively (Figure 1B).

ECL-induced relaxation could be due to a direct action on the smooth muscle cells or to an indirect action mediated by the endothelium as is now well established for ACh and other agents.^5^ To distinguish between these alternatives, the effect of ECL was determined in rabbit thoracic aorta rings containing endothelium or in rings in which the endothelium had been removed, as can be observed in figure 1C. ECL did not cause relaxations in rings without endothelium.

In order to determine whether ECL caused EDR in other arteries from the rabbit or in arteries from other animal species, the effect of ECL was determined in rings from rabbit abdominal aorta, rabbit superior mesenteric artery, rabbit common carotid artery, rat thoracic aorta, guinea-pig thoracic aorta, mouse thoracic aorta, and porcine coronary arteries. As shown in figure 1D and 1E, ECL at 1.5µg/mL caused relaxations which were also strictly endothelium-dependent in rat thoracic aorta and in porcine right coronary artery rings. Similar findings were observed in all the other arteries tested.

**Figure 2:**
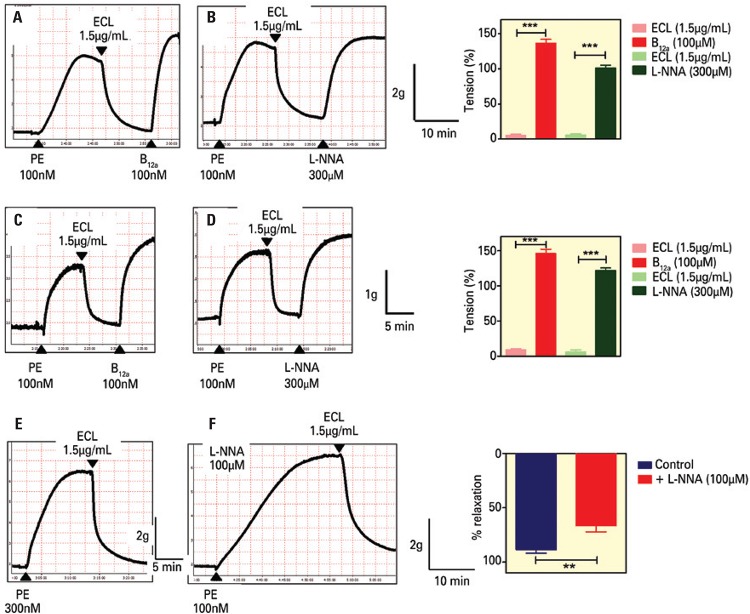
Representative tracings showing the reversion of extract of *Combretum leprosum*-induced (ECL-induced) relaxations by hydroxocobalamin (B12a; 100µM,) or L-NG-nitro-L-arginine (L-NNA; 300µM) on rings of rabbit thoracic aorta (A, B) or rat thoracic aorta (C, D). Representative tracings showing ECL-induced relaxations in the absence (E; n=8) and in the presence of L-NNA (F; 100µM, n=7) in rabbit abdominal aortic rings. Right panels (A-D) show the mean ± standard error of the mean (SEM) values of tension of 4-8 similar experiments (tension expressed as a percentage of phenylephrine - PE-induced contraction). **Two-tailed p=0.0041 (unpaired *t*-test); ***two-tailed p<0.0001 (paired *t*-test). Right panel (E-F) shows the mean ± SEM values of the magnitude of ECL-induced relaxation (relaxation magnitude, expressed as a percentage of reduction of PE-induced contraction)

In rabbit and rat thoracic aorta, EDR are entirely mediated by NO; thus, the effect of a nitric oxide synthase (NOS) inhibitor (L-NNA) and of a NO scavenger (B_12a_) upon ECL- induced EDR was examined. As shown in figures 2A, 2B, 2C, 2D, and 2E the addition of L-NNA or B_12a_ on the plateau of ECL-induced relaxations of rabbit and rat aortic rings completely reversed the relaxations. ECL (1.5µg/mL) was also able to induce relaxations in rabbit abdominal aorta in the presence of L-NNA (100µM, 20 minutes) (Figure 2F). The magnitude of these relaxations was smaller than the ones observed in the absence of L-NNA.

**Figure 3:**
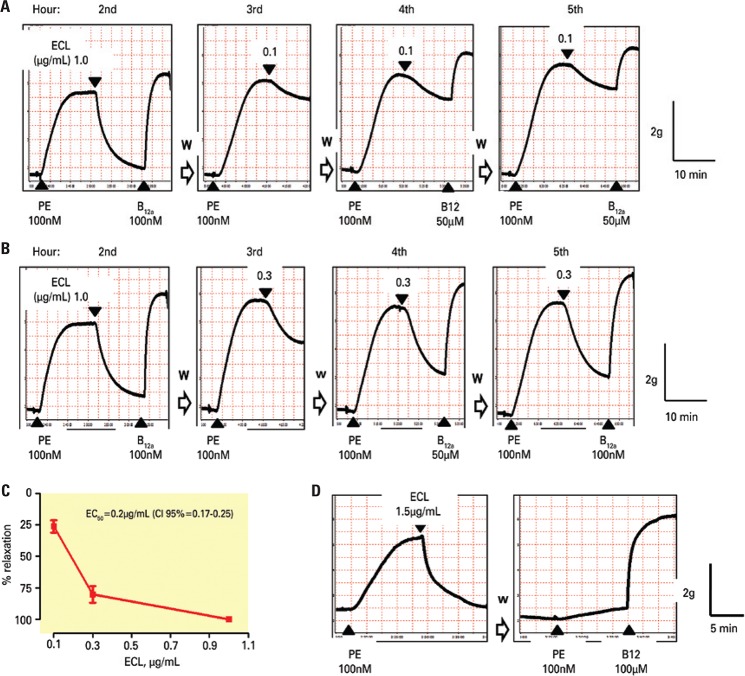
Representative tracings showing the effect of low concentrations of extract of *Combretum *leprosum (ECL) on rings from rabbit thoracic aorta pre-constricted with phenylephrine (PE). The small concentrations of ECL were added in a non-cumulative manner after “priming” of preparations with ECL (1.0µg/mL) and reversion of ECL-induced relaxations by hydroxocobalamin (B12a; 100µM). It can be observed that the magnitude of ECL (0.1-0.3µg/mL)-induced relaxations increased in the first additions and became stabilized only after the 4th or 5th hour (A and B). Panel C shows the concentration-effect curve (mean ± standard error of the mean - SEM values) observed after the 4 hours (**two-tailed p<0.0067, paired *t*-test). Panel D shows that the magnitude of PE-induced contraction was reduced even removing ECL (1.5µg/mL) from the chamber and the recovery of contraction by B12a addition. W= washout of the preparation for 60-45 minutes; EC50: half-maximal relaxation; 95%CI: 95% confidence interval

In experiments designed to calculate the potency of the extract by the single concentration method using the same rabbit thoracic aorta ring, it was observed that the magnitude of the relaxation induced by low concentrations (0.1 to 0.3µg/mL) of ECL increased with successive additions and required at least three additions of the same concentration at hourly intervals in order to become stabilized (Figures 3A and 3B); furthermore it was observed that an initial priming of the rings with 1µg/mL was also required. In these conditions the calculated EC_50_ was 0.2µg/mL (95% confidence interval − 95%CI=0.17-0.25) (Figure 3C).

Interestingly, the magnitude of contractions elicited by PE after washing out the extract, especially at the higher concentrations (1 to 1.5µg/mL), did not return to that observed before adding the extract. For example, the magnitude of PE-induced contractions 1 hour after washing out the ECL (1.5µg/mL) was 0.62±0.11g (n=4), which is significantly lower than 2.22±0.15g (n=4) observed before adding ECL. Furthermore, the magnitude of PE-induce contractions remained reduced (1.17±0.14g; n=4) even 4 hours after washing out ECL, whereas the addition of B_12a _increased the magnitude of PE-induced contraction (which was decreased 1 hour after ECL washout) from 0.30±0.04g (n=4) to 3.70±0.21g (n=4) (Figure 3D).

**Figure 4:**
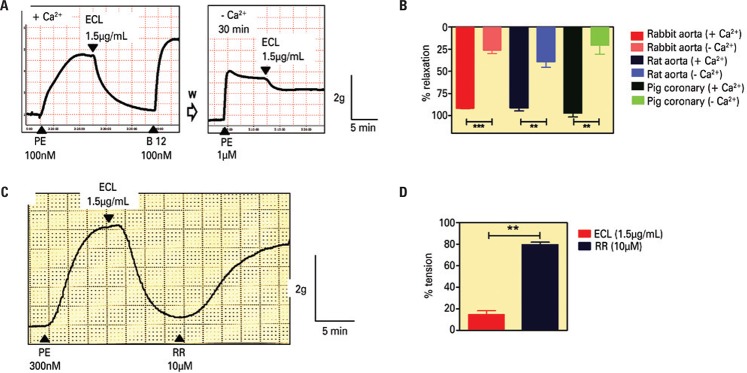
Representative tracings showing the effect of extract of *Combretum *leprosum (ECL; 1.5µg/mL) on the isometric tension developed by rings of rabbit thoracic aorta pre-constricted with phenylephrine (PE), in A, in the presence (+ Ca2+) or absence (- Ca2+) of extracellular Ca2+. In B, the mean ± standard error of the mean - SEM values of the magnitude of relaxation (relaxation magnitude expressed as a percentage reduction of PE- or U46619-induced contraction) of four similar experiments in rings from thoracic rabbit aorta, thoracic rat aorta, and right coronary pig artery. In C, the reversion of ECL-induced relaxations by ruthenium red (RR, 10µM) and D shows the mean ± SEM values of tension of three similar experiments (tension expressed as a percentage PE-induced contraction). **Two-tailed p<0.001 or ***p<0.0001 (paired *t*-test). W: washout of the preparation for 60 minutes; -Ca2+: washing and incubation with Krebs solution for 30 minutes

Considering that endothelial cells require the presence of Ca^2^ in the extracellular medium in order to produce NO and EDHF (endothelium-derived hyperpolarization factor), we determined whether the effect of ECL was affected by absence of extracellular Ca^2^. As shown in figures 4A and 4B, when rabbit thoracic aorta, rat thoracic aorta, and pig right coronary artery rings were incubated in Krebs solution without Ca^2^ (-Ca^2^), the magnitude of ECL-induced relaxations was significantly reduced. In order to identify pharmacologically the putative Ca^2^ influx pathways activated by ECL, the effect of RR was next examined. In rabbit thoracic aorta rings, the addition of RR (10µM) on the plateau of ECL-induced relaxations reversed almost completely such relaxations (Figures 4C and 4D). Similar results were observed in rings from rat and mice thoracic aorta.

Because tree barks are rich in polyphenol compounds, the amount of total polyphenols present in the ECL was estimated using the FC method using GA as standard of polyphenol-like reactive compounds. From the linear regression equation relating absorbance and gallic acid concentration (y=0.0578 x -0.0348), it was calculated that a solution of 12.5mg/mL of ECL contained 4.94mg/mL (approximatively 39.5%) of gallic acid equivalents.

## DISCUSSION

The present study showed for the first time that ECL produced relaxations of isolated arterial rings from different animal species, which indicated that the bark of *C. leprosum* contains bioactive compounds capable of influencing the function of blood vessels. Extracts or isolated compounds of *C. leprosum* have been previously shown to exhibit antinociceptive,^8-11^ anticholinesterase,^12^ antiulcerogenic,^13^ antileishmanial,^14^ anti-inflammatory, and antiproliferative effects;^15,16^ however, to our knowledge, the vascular effects described in the present work have not been mentioned previously.

The fact that the effect was observed at relative low concentrations (0.1 to 1.0µg/mL), and, considering that it is crude extract that may contain hundreds of different compounds, indicates that the bioactive compound responsible for the effect is either the most abundant, or if present in small amounts, is extremely potent; alternatively the effect may be caused by the synergistic action of different compounds. Such synergism has been described for polyphenols present in red wine extract (RWP), for instance, the EC_50_ of raw RWP to induce EDR in rat aorta is 0.53 to 0.63µg/mL, while the EC_50_ of pure delphinidin or leucocyanidol, polyphenol compounds isolated from RWP, are 8.91µg/mL and 1.77µg/mL, respectively.^17,18^ Substances identified in other *Combretum *genera members are also capable of inducing EDR in rat aortas; however the concentrations required are higher than those of ECL; for example, 5 to 80µg/mL (to mollic acid glucoside isolated from *C. molle* leaf) and EC_50_ of 3.9µg/mL and 9.5µg/mL for methanolic extract of *C. celastroides* leaves, and *C. racemosum* roots respectively.^3,4^ As in the rat aorta, 1.5µg/mL of ECL causes complete relaxation of PE-induced contractions the vasodilator in the extract of compounds present in ECL are unlikely to be related to those already described in other members of the genus *Combretum*.

The endothelial cells might constitute the main site of action of the bioactive compounds present in the ECL, this suggestion is based on the observation that it requires an intact endothelium to cause relaxation in all the arteries examined. Alternatively, the ECL could be acting on the vascular smooth cells to enhance their responsiveness to relaxing factors produced basally by the endothelium like as the phosphodiesterase inhibitors but the relaxations caused by these inhibitors is still present in endothelium denuded rings from rat or rabbit thoracic aorta.^19,20^ In fact, such mechanism has been recently proposed for the relaxant effect of a dichloromethane fraction from *Anogeissus leiocarpus*, a combretaceae.^21^

Considering that ECL-relaxed thoracic aorta rings from both rabbits and rats in which BK has no effect^6^ and that histamine relaxes rat thoracic aorta but not rabbit thoracic aorta,^22^ together with the fact that it relaxes rings from pig coronary arteries in which ACh causes contraction would suggest that the mechanisms involved in its action are different from those involved in the action of classical receptor agonists which cause EDR. In addition, the kinetic characteristics of ECL-induced relaxation such as slower initiation and longer time for attaining the steady state clearly distinguish its effects from those caused by ACh and BK and histamine. Furthermore, the fact that the effect remained for at least 4 hours after removing the extract contrasts with the transient relaxation caused by ACh or BK whose effects are not already evident 0.5 to 1 hour after removing them from the organ bath.

As in some arteries (rabbit superior mesenteric and common carotid arteries, as well as from pig coronary artery and from guinea-pig thoracic aorta) ECL is able to cause EDR in the presence of NOS inhibitors, we suggest that its effect is apparently related to the activation of endothelial cells to produce whatever relaxing factor is being predominantly produced. It has been thoroughly described that endothelial cells are heterogeneous in terms of responsiveness to agonists and relaxing factors being produced; for instance, endothelial cells from small arteries and arterioles produce mainly EDHF, whereas endothelial cells from the aorta produce almost exclusively NO.^23^

Our results showing that a major part of ECL-induced EDR requires the presence of extracellular Ca^2^ indicate that ECL action is related to the activation of Ca^2^ influx in endothelial cells. Although the pathways mediating Ca^2^ influx in endothelial cells have not been yet entirely elucidated, the fact that RR was able to revert ECL-induced relaxations would suggest that one or more of the various RR-sensitive Ca^2^ permeable channels^24^ is activated directly or indirectly by bioactive compounds in the extract.

There are good reasons to suggestion that polyphenol compounds could constitute the active principles accounting for the ECL effect. First, substances with polyphenol-like chemical reactivity are present in ECL in substantial amounts (approximately 40% w/w). Second, plant-derived polyphenols have been shown to cause EDR in several arterial segments.^25^ Third, previous studies have shown the presence of a variety of compounds, including polyphenols, in the ethanol extracts of flowers, leaves, roots, fruits, and bark from *C. leprosum*, such as flavonoids,^12,26,27^ triterpenes,^26-28^ mono- and oligosaccharides, fatty acids, sterols,^12,27,28^ and stilbenes.^29^

Finally the description of long-lasting vasorelaxing effect of ECL might be clinically relevant since it could lead to the development of potential new therapeutic approaches for pathological conditions in which blood flow is reduced or impaired due to increased vascular tonus such as hypertension and vasoespastic disorders. More experiments are necessary to establish the therapeutic potential of ECL, for instance we need to answer the questions: What are effects of ECL upon blood circulation in intact animals? What are the active compounds present in the extract? What are the mechanisms by which the ECL promotes synthesis and release of endothelial factors?

## CONCLUSION

The present findings showed for the first time that an aqueous suspension of a lyophilized hydroalcoholic extract of *Combretum leprosum* causes relaxation of arterial rings isolated from different animal species. These *Combretum leprosum* extract-induced relaxations were completely dependent on a functional endothelium and appear to be mediated by the release of endothelial relaxing factors such as nitric oxide in the rat or rabbit thoracic aorta or by nitric oxide and others factors non-derived from nitric oxide synthases or cyclooxygenase activity in rabbit abdominal aorta and pig coronary arteries. Finally, since *Combretum leprosum* extract requires Ca^2^ influx through ruthenium red-sensitive pathways to cause relaxation the elucidation of its mechanism of action, this could reveal a new approach to activate endothelial cells to produce and release relaxing factors.
