# Supplementary material for: Long-lasting endothelium-dependent relaxation of isolated arteries caused by an extract from the bark of Combretum leprosum
Source: Einstein (Sao Paulo). 2015 Jul-Sep;13(3):395–403. doi: 10.1590/S1679-45082015AO3242 (PMC4943785; doi:10.1590/S1679-45082015AO3242)
Supplement: Translation

# Translation: Extrato das cascas de *Combretum leprosum* causa relaxamento dependente de endotélio de longa duração em artérias isoladas

**Authors:** Francisco das Chagas Alves, Paulo Marques da Silva Cavalcanti, Rita de Cassia Aleixo Tostes Passaglia, Gustavo Ballejo

**Affiliations:** 1 Faculdade de Medicina de Ribeirão Preto, Universidade de São Paulo, Ribeirão Preto, SP, Brasil.; 2 Universidade Federal do Piauí, Teresina, PI, Brasil.

**Keywords:** Combretum, Artérias/efeitos de drogas, Endotélio/efeitos de drogas, Óxido nítrico/farmacologia, Polifenóis/farmacologia

## Abstract

**Objetivo:**

Descrever e caracterizar os relaxamentos induzidos por um extrato das cascas de *Combretum leprosum* em anéis de artérias de diferentes espécies de animais.

**Métodos:**

Anéis (3 a 4mm) de artérias de coelho, rato e porco foram montados em cubas para órgão isolado (Krebs, 37°C, 95%O_2_/5%CO_2_) para registro das contrações isométricas. Após um período de estabilização (2 a 3 horas), as contrações foram induzidas com fenilefrina (0,1 a 0,3µM) ou U46619 (10 a 100nM); no platô dessas contrações, adicionamos o extrato* Combretum leprosum*. Diferentes protocolos foram realizados para determinar potência, duração, reversibilidade e mecanismo dos relaxamentos induzidos pelo extrato.

**Resultados:**

Em todas as preparações testadas, o extrato de *Combretum leprosum* (1,5µg/mL) provocou relaxamentos dependentes de endotélio. Em aorta torácica de coelho ou rato, os relaxamentos foram revertidos pela vitamina B_12_a ou L-NG-nitro-arginina. Em anéis de aorta abdominal de coelho e de artérias coronárias de porco, o extrato causou relaxamentos sensíveis e resistentes à L-NG-nitro-arginina. Em aorta torácica de coelho, o extrato foi relativamente muito potente (EC_50_=0,20μg/mL) e quando causou relaxamentos; intrigantemente o endotélio continuou a produzir fatores relaxantes por um longo período após remoção do extrato. A magnitude dos relaxamentos induzidos pelo extrato foi significativamente reduzida em ausência Ca^2+^ extracelular; ademais, o vermelho de rutênio (10μM), um bloqueador de canais TRPs, foi capaz de reverter os relaxamentos induzidos pelo extrato. Análises preliminares indicaram que o extrato continha compostos com reatividade química semelhante à polifenóis.

**Conclusão:**

O extrato de *Combretum leprosum* contem compostos bioativos capazes de promover estimulação dependente de Ca^2+^ das células endoteliais a qual resulta numa produção prolongada de fatores relaxantes.

## INTRODUÇÃO

*Combretum leprosum *Mart. (*Combretaceae*) é um arbusto ou pequena árvore que cresce no Nordeste do Brasil, onde é conhecido como mufumbo, mofumbo, cipoaba e pente-de-macaco. Na medicina popular, os extratos de diferentes partes da planta são utilizados por suas propriedades supostamente expectorantes, hemostáticas, sedativas e afrodisíacas.^1,2^

Em uma triagem baseada na bioatividade dos possíveis efeitos do extrato hidroalcoólico liofilizado da casca de *Combretum leprosum* (ECL) em músculos lisos viscerais e vasculares, observou-se que o extrato causou um potente relaxamento dependente do endotélio (*endothelium-dependent relaxation* - EDR)dos anéis da aorta torácica de coelho. Embora tenha sido descrito que substâncias isoladas de espécies relacionadas do gênero *Combretum* causam EDR na aorta de ratos (glicosídeo ácido móllico isolado do *Combretum molle*), bem como frações metanólicas ricas em flavonoides de *Combretum celastroides* e *Combretum racemosum*,^3,4^ todos os efeitos de extratos de flores, folhas e/ou raízes de ECL previamente descritos não incluem nenhuma bioatividade cardiovascular.

As células endoteliais exibem uma heterogeneidade notável na resposta a agentes que induzem ao EDR seja de artérias homólogas de diferentes espécies animais ou de artérias de diferentes leitos vasculares do mesmo animal; por exemplo, a acetilcolina (ACh), mas não a bradicinina (BK), induz ao EDR na aorta de coelho e de rato;^5,6^ de forma semelhante, a histamina causa EDR na aorta de rato, mas não na de coelho. Assim, ao descrever o EDR de uma nova droga ou produto natural, é altamente recomendado analisar seus efeitos na mesma artéria em mais de uma espécie animal e em diferentes artérias do mesmo animal.

## OBJETIVO

Descrever e interpretar os resultados de experimentos concebidos para responder às seguintes perguntas relacionadas ao novo efeito observado do extrato de *Combretum leprosum*: (1) Qual o local de ação, a potência e a duração do efeito do extrato de *Combretum leprosum* para provocar relaxamentos dos anéis da aorta torácica de coelho? (2) O extrato de *Combretum leprosum* é capaz de causar relaxamento dependente do endotélio em anéis arteriais isolados de outros vasos de coelho ou de vasos de rato, camundongo, cobaia e porco? (3) Quais os possíveis mecanismos envolvidos no relaxamento induzido por extrato de *Combretum leprosum* dos anéis da aorta torácica de coelho?

## MÉTODOS

### Material vegetal

A casca do tronco de *C. leprosum* foi coletada pela manhã (entre 10 e 11h) no dia 15 de julho de 2005 no Centro de Ciências Agrárias da Universidade Federal do Piauí, em Teresina (PI), Brasil. Uma amostra (número 10.557) foi depositada no Herbário Graziela Barroso (TEPB, na mesma instituição). O material vegetal passou por secagem à sombra a 40±1ºC, e o pó da casca do tronco (500g) passou por extração (três vezes) com 1L de etanol a 70%. O extrato hidroalcoólico foi evaporado em vácuo a 50°C e liofilizado para a obtenção de um extrato seco, que foi armazenado sob refrigeração (4°C) até o uso. O extrato foi diluído novamente em água destilada para os experimentos.

### Experimentos com órgãos isolados

Trinta coelhos neozelandeses machos e fêmeas (2,5 a 3,5kg), 20 ratos *Wistar* (200 a 250g), 5 cobaias inglesas (350g) e 10 camundongos machos (24 a 30g) foram utilizados em todos os experimentos. Os animais foram obtidos junto ao biotério do Campus de Ribeirão Preto da Universidade de São Paulo. Os corações de dez porcos foram obtidos de um abatedouro local, tendo sido removidos imediatamente após o óbito do animal, lavados com solução de Krebs fria para remover a maior parte do sangue, e depois transportados para o laboratório em sacos plásticos lacrados acondicionados em um isopor com gelo picado. Duas a três horas após a remoção do coração dos animais, as artérias coronárias direita e/ou esquerda foram dissecadas para preparação dos anéis arteriais. Todos os protocolos experimentais seguiram os princípios éticos para experimentação em animais recomendados pelo Colégio Brasileiro de Experimentação Animal e foram aprovados pelo Comitê de Ética em Experimentação Animal (número 084/2011) da Faculdade de Medicina de Ribeirão Preto da Universidade de São Paulo.

Os ratos, camundongos, cobaias e coelhos foram sacrificados utilizando anestesia com pentobarbital sódio (50mg/kg i.p. para os ratos, camundongos e cobaias e/ou por injeção na veia marginal da orelha nos coelhos). A aorta torácica descendente (de todos os animais), além da aorta abdominal, da artéria mesentérica superior e da artéria carótida comum (dos coelhos), foi rapidamente removida e colocada em solução de Krebs (116mM de cloreto de sódio − NaCl; 4,5mM de cloreto de potássio − KCl; 1,14mM de fosfato monossódico − NaH_2_PO_4_; 1,16mM de cloreto de magnésio − MgCl_2_; 2,5mM de cloreto de cálcio − CaCl_2_; 25mM de bicarbonato de sódio − NaHCO_3_; e 11,1nM de D-glicose) contendo diclofenaco (10µM) para inibir a síntese de produtos derivados da ciclo-oxigenase.^6^ Após a remoção de todos os tecidos aderentes, as artérias foram seccionadas em anéis (3 a 4mm), com cuidado para preservar o endotélio. Quando necessário, esfregou-se a túnica íntima de alguns dos anéis com uma varinha de aço para destruir o endotélio. Os anéis foram suspensos entre suportes metálicos finos e colocados sob uma tensão inicial de 2 a 4g em cubas para órgão isolado (volume: 5 a 10mL) contendo solução de Krebs a 37±1°C borbulhado continuamente com 5% de dióxido de carbono (CO_2_) − 95% de oxigênio (O_2_). A tensão isométrica de cada anel foi registrada continuamente e arquivada utilizando o sistema de aquisição de dados *Lab Chart*.

### Protocolos experimentais

Para testar a viabilidade dos anéis, concentrações submáximas de fenilefrina (*Phenylephrine* - PE) (PE; 0,1 a 1μmol/L) ou U46619 (10 a 30nmol/L) foram adicionadas duas vezes, de hora em hora. Durante o platô de contração induzido pela segunda adição de PE (ou U46619), a integridade funcional do endotélio foi testada mediante a adição de ACh (1μmol/L) ou BK (0,1μmol/L, artéria coronária de porco). Os anéis nos quais a ACh (ou a BK) causou uma queda de ao menos 80% da tensão induzida por PE (ou U46619) foram considerados como contendo endotélio funcional intacto. Uma hora após a determinação da funcionalidade do endotélio, foi novamente adicionada PE (ou U46619) para a contração dos anéis, e uma concentração supramáxima de ECL (1,5μg/mL) foi adicionada.

Para determinar se a síntese e/ou a liberação de óxido do nítrico (*Nitric Oxide* - NO) eram necessárias para o relaxamento induzido por ECL, L-N^G^-nitro-L-arginina (L-NNA; 300μmol/L) ou hidroxocobalamina (B_12a_; 30 a 100μM) foram adicionadas quando o relaxamento induzido por ECL atingiu um patamar estável. Para determinar se o relaxamento induzido por ECL requeria a presença de Ca^2^extracelular, o efeito do ECL foi testado em preparações incubadas em solução de Krebs contendo nominalmente zero Ca^2^. Para determinar se o influxo de Ca^2^ nas células endoteliais estava envolvido no relaxamento induzido por ECL, foi determinado o efeito da adição de vermelho de rutênio *ruthenium red *- RR; 10μmol/L, um bloqueador não seletivo dos canais permeáveis de Ca^2^) quando a resposta de relaxamento induzida por ECL atingiu a estabilidade também.

A potência do ECL foi calculada a partir de curvas concentração-efeito obtidas de forma não cumulativa utilizando anéis de aorta torácica de coelho com endotélio intacto, isto é, foi adicionada uma única concentração de ECL (0,1, 0,3 e 1,0µg/mL) por vez no platô das contrações induzidas por PE em intervalos de 45 a 60 minutos. Todas as drogas e as soluções de extratos foram adicionadas no meio das cubas com uma pipeta num volume de 1 a 30µL.

### Mensuração dos polifenóis

Para estimar os grupos fenólicos OH totais, foi utilizado o método Folin-Ciocalteu (FC).^7^ As soluções de ECL (0,5mg/mL) foram diluídas em hidróxido de sódio (NaOH) aquoso (1%) para produzir soluções com as seguintes concentrações: 1,0, 2,5, 5,0, 7,5, 10, e 12,5µg/mL. A essas soluções, foram adicionados 500µL de reagente FC, o que foi seguido de agitação vigorosa por 2 minutos. Depois, foram adicionados 500µL de carbonato de sódio (Na_2_CO_3_; 75g/mL), e essas soluções foram incubadas a temperatura ambiente ou a 50°C, por 20 minutos. As incubações foram finalizadas mediante resfriamento das soluções pela imersão em banhos de gelo por 5 minutos. A absorbância específica de 700nm foi determinada utilizando uma leitora de placas de *Enzyme Linked Immuno Sorbent Assay* (ELISA); as análises foram realizadas em triplicado. Soluções de ácido gálico (AG) e quercetina (dissolvida em água contendo 10% metanol) foram utilizadas como compostos polifenóis padrão.

### Análises estatísticas

O relaxamento induzido por ECL em anéis arteriais foi expresso como a porcentagem de redução (% de tensão) da tensão desenvolvida por contrações induzidas por PE ou U46619. A reversão desse relaxamento por inibidores foi expressa como porcentagem da magnitude de relaxamento (% de relaxamento). Os valores foram apresentados como média ± erro padrão da média (EPM). A concentração de ECL que causa a metade do relaxamento máximo (EC_50_) foi determinada pelo ajuste da curva concentração-resposta original a uma curva sigmoide utilizando o programa *GraphPad Prism* ^*®*^ *Software*, versão 5.0. Os valores (em porcentagem) das mudanças de tônus foram analisados pelo teste *t* de *Student* (bicaudal), pareado (no mesmo anel arterial) ou não pareado (em anéis arteriais diferentes). Os valores de p<0,05 foram considerados estatisticamente significativos.

### Substâncias químicas

ACh, BK, reagente de FC, AG, B_12a_, PE, quercetina, etanol de grau reagente, RR e U46619 foram obtidos da Sigma (Estados Unidos). O diclofenaco foi obtido da *Calbiochem* ^*®*^ (Estados Unidos). A L-NNA foi obtida da *Research Biochemicals International* (Estados Unidos).

## RESULTADOS

Experimentos iniciais utilizando anéis da aorta torácica de coelhos com endotélio intacto mostraram que uma suspensão aquosa de ECL na concentração de 1,0 a 1,5µg/mL causou relaxamentos de magnitude semelhante ou até maiores do que os causados pela Ach (1µM) (Figura 1A). Enquanto os relaxamentos provocados pela ACh tiveram início quase imediatamente após sua adição, aqueles provocados pelo ECL precisaram de 60 a 90 segundos até se tornarem aparentes; ademais, enquanto o efeito relaxante da ACh atingiu um platô em aproximadamente 2 minutos, os efeitos induzidos pelo ECL levaram aproximadamente 10 minutos para atingir o platô. A velocidade máxima do relaxamento, calculada a partir da primeira derivada (dT/dt), foi de -90,33±5,41mg/s (n=8) e de -39,83±2,28mg/s (n=8) para a ACh (1µM) e o ECL (1,0µg/mL), respectivamente (Figura 1B).

**Figura 1:** Traçados representativos mostrando o efeito da acetilcolina (ACh; 1µM) e do extrato de *Combretum leprosum* (ECL; 1,0µg/mL) sobre a tensão isométrica desenvolvida pelos anéis da aorta torácica de coelho pré-contraídos com fenilefrina (PE; 100nM) (A). Primeira derivada (dT/ dt, mg/s) dos relaxamentos induzidos por ECL (1,0µg/mL) e ACh (1µM) (B). Efeito do ECL (1,5µg/mL) sobre a tensão isométrica desenvolvida pelos anéis, com (+E) ou sem (-E) o endotélio, da aorta torácica de coelho (C), da aorta torácica de rato (D) pré-contraída com PE, e da artéria coronária direita de suínos pré-contraída com U46619 (E). Em (F), valores médios ± erro padrão da média (EPM) da magnitude do relaxamento observado em 4-20 experimentos semelhantes (a magnitude de relaxamento é expressa como porcentagem da magnitude das contrações induzidas por PE ou U46619). ***Teste bicaudal p<0,0001 (teste *t* não pareado). L: lavagem da preparação por 60 a 45 minutos

O relaxamento induzido por ECL pode ser devido a uma ação direta sobre as células musculares lisas ou a uma ação indireta mediada pelo endotélio, como já está bem estabelecido para a ACh e outros agentes.^5^ Para distinguir entre essas alternativas, o efeito do ECL foi determinado em anéis da aorta torácica de coelho contendo endotélio ou em anéis nos quais o endotélio foi removido, como pode ser observado na figura 1C, que mostra que o ECL não causou relaxamento em anéis sem endotélio.

Para determinar se o ECL causa EDR em outras artérias de coelho ou em artérias de outras espécies animais, o efeito do ECL foi determinado em anéis da aorta abdominal de coelho, da artéria mesentérica superior de coelho, da artéria carótida comum de coelho, da aorta torácica de rato, da aorta torácica de cobaia, da aorta torácica de camundongo e das artérias coronárias de suínos. Conforme apresentado nas figuras 1D e 1E, o ECL a 1,5µg/mL causou relaxamentos que também foram estritamente dependentes do endotélio nos anéis da aorta torácica de rato e da artéria coronária direita de suínos. Achados semelhantes foram observados em todas as outras artérias testadas.

Na aorta torácica de coelho e de rato, os EDR são inteiramente mediados por NO; portanto, foi analisado o efeito de um inibidor de síntase de óxido nítrico (*NitricOxide Synthase* - NOS) (L-NNA) e de um sequestrador de NO (B_12a_) sobre os EDR induzidos por ECL. Conforme apresentado nas figuras 2A a 2E, a adição de L-NNA ou de B_12a_ no platô do relaxamento induzido por ECL nos anéis de aorta de coelho e rato reverteu completamente o relaxamento. O ECL (1,5µg/mL) também foi capaz de induzir relaxamentos na aorta abdominal de coelho na presença de L-NNA (100µM por 20 minutos) (Figura 2F). A magnitude desses relaxamentos foi menor do que aqueles observados na ausência de L-NNA.

**Figura 2:** Traçados representativos mostrando a reversão de relaxamento induzido por extrato de *Combretum leprosum* (ECL) por hidroxocobalamina (B12a; 100µM) ou L-NG-nitro-L-arginina (L-NNA; 300µM) nos anéis da aorta torácica de coelho (A, B) ou de rato (C, D). Em (E e F), traçados representativos mostrando o relaxamento induzido por ECL na ausência (E; n=8) e na presença de L-NNA (F; 100µM, n=7) em anéis da aorta abdominal de coelho. Os painéis do lado direito (A-D) mostram os valores médios ± erro padrão da média (EPM) da tensão de 4-8 experimentos semelhantes (tensão expressa como uma porcentagem da contração induzida por fenilefrina - PE). **Teste bicaudal p=0,0041 (teste *t* não pareado); ***teste bicaudal p<0,0001 (teste *t* pareado). O painel do lado direito (E-F) mostra os valores médios ± EPM do relaxamento induzido por ECL (magnitude do relaxamento expresso como porcentagem da contração induzida por PE)

Em experimentos planejados para calcular a potência do extrato por meio do método não cumulativo utilizando o mesmo anel da aorta torácica de coelho, observou-se que a magnitude do relaxamento induzido por baixas concentrações (0,1 a 0,3µg/mL) de ECL aumentou com adições sucessivas e foram necessárias ao menos três adições da mesma concentração a cada hora para atingir a estabilidade (Figuras 3A e 3B); ademais, observou-se que uma pré-ativação inicial dos anéis com 1µg/mL também foi necessária. Nessas condições, o EC_50_ calculado foi de 0,2µg/mL (intervalo de confiança de 95% − IC 95%=0,17-0,25) (Figura 3C).

**Figura 3:** Traçados representativos mostrando o efeito de baixas concentrações de extrato de *Combretum leprosum* (ECL) sobre os anéis da aorta torácica de coelho pré-contraídos com fenilefrina (PE). As concentrações de ECL foram adicionadas de forma não cumulativa após a “ativação” das preparações com ECL (1,0µg/mL) (A), assim como a reversão do relaxamento induzido por ECL por hidroxocobalamina (B12a; 100µM). Pode-se observar que a magnitude do relaxamento induzido por ECL (0,1-0,3µg/mL) aumentou progressivamente com as adições sucessivas e se estabilizou somente após a quarta ou quinta hora (A e B). O painel C mostra a curva de concentração-efeito (valores médios ± erro padrão da média - EPM) observada após as 4 horas (**teste bicaudal p<0,0067, teste *t* pareado). Em D, um traçado representativo mostrando que a magnitude das contrações induzidas por PE permaneceu reduzida mesmo 1 hora após a remoção do ECL (1,5µg/mL) da cuba e a recuperação da contração por meio da adição de B12a. L: lavagem da preparação por 60 a 45 minutos; EC50: metade do relaxamento máximo; IC95%: intervalo de confiança de 95%

Curiosamente, a magnitude das contrações provocadas por PE após a lavagem do extrato, especialmente nas concentrações mais altas (1 a 1,5µg/mL), não retornou aos níveis observados antes da adição do extrato. Por exemplo, a magnitude das contrações induzidas por PE 1 hora após a lavagem do ECL (1,5µg/mL) foi de 0,62±0,11g (n=4), que é significativamente menor do que os 2,22±0,15g (n=4) observados antes da adição de ECL. Além disso, a magnitude da contração induzida por PE permaneceu reduzida (1,17±0,14g; n=4), mesmo 4 horas após a lavagem do ECL, enquanto a adição de B_12a _aumentou a magnitude da contração induzida por PE (a qual estava diminuída 1 hora após a lavagem do ECL) de 0,30±0,04g (n=4) para 3,70±0,21g (n=4) (Figura 3D).

Considerando que as células endoteliais requereram presença de Ca^2^ no meio extracelular para produzir NO e fator hiperpolarizante derivado do endotélio (*endothelium-derived hyperpolarizing factors* - EDHF) determinamos se o efeito do ECL era afetado pela ausência de Ca^2^extracelular. Conforme apresentado nas figuras 4A e 4B, quando os anéis da aorta torácica de coelho, da aorta torácica de rato e da artéria coronária direita de porco foram incubados em uma solução de Krebs sem Ca^2^ (- Ca^2^), a magnitude dos relaxamentos induzidos por ECL foi reduzida significativamente. A fim de identificar farmacologicamente as possíveis vias de influxo de Ca^2^ ativadas pelo ECL, foi analisado, a seguir, o efeito do RR. Nos anéis da aorta torácica de coelho, a adição de RR (10µM) no platô do relaxamento induzido por ECL reverteu quase totalmente esse relaxamento (Figuras 4C e 4D). Resultados semelhantes foram observados em anéis da aorta torácica de ratos e camundongos.

**Figura 4:** Traçados representativos mostrando o efeito do extrato de *Combretum leprosum* (ECL; 1,5µg/mL) sobre a tensão isométrica desenvolvida pelos anéis da aorta torácica de coelho pré-contraídos com fenilefrina (PE) (A), na presença (+Ca2+) ou ausência (-Ca2+) de Ca2+ extracelular. Em B, valores médios ± erro padrão da média (EPM) da magnitude do relaxamento (magnitude do relaxamento expresso como porcentagem da redução da contração induzida por PE ou U46619) de quatro experimentos semelhantes em anéis da aorta torácica de coelho e da artéria coronária de porco. Em C, reversão do relaxamento induzido por ECL pelo vermelho de rutênio (RR, 10µM) e, em D, os valores médios ± EPM da tensão de três experimentos semelhantes (tensão expressa como porcentagem da contração induzida por PE). **Teste bicaudal p<0,001 ou ***p<0,0001 (teste *t* pareado). L: lavagem da preparação por 60 minutos; -Ca2+: lavagem e incubação com solução de Krebs por 30 minutos

Como as cascas de árvore são ricas em compostos polifenóis, a quantidade total de polifenóis presente no ECL foi estimada utilizando-se o método de FC, empregando o ácido gálico como o padrão de compostos polifenólicos. A partir da equação de regressão linear relacionando a absorbância e a concentração de ácido gálico (y=0,0578 x -0,0348), calculou-se que uma solução de 12,5mg/mL de ECL continha 4,94mg/mL (aproximadamente 39,5%) de equivalentes de ácido gálico.

## DISCUSSÃO

O presente estudo mostrou, pela primeira vez, que o ECL produziu relaxamentos de anéis arteriais isolados de diferentes espécies animais, o que indicou que a casca de *C. leprosum* contém compostos bioativos capazes de influenciar a função de vasos sanguíneos. Demonstrou-se anteriormente que extratos ou compostos isolados de *C. leprosum* exibiram efeitos antinociceptivos,^8-11^ anticolinesterásicos,^12^ antiulcerogênicos,^13^ antileishmania,^14^ anti-inflamatórios e antiproliferativos;^15,16^ no entato, até onde sabemos, os efeitos vasculares descritos no presente trabalho não foram mencionados anteriormente.

O fato de que o efeito foi observado em concentrações relativamente baixas (0,1 a 1,0µg/mL) e, considerando que se trata de um extrato bruto que pode conter centenas de diferentes compostos, trata-se de uma indicação de que o composto bioativo responsável pelo efeito é o mais abundante ou, quando presente em pequenas quantidades, é extremamente potente; por outro lado, o efeito pode ser causado também pela ação sinérgica de diferentes compostos. Tal sinergismo tem sido descrito para os polifenóis presentes no extrato de vinho tinto (RWP), por exemplo, o EC_50_ dos RWP brutos para induzir ao EDR na aorta do rato é de 0,53 a 0,63µg/mL, enquanto o EC_50_ da delfinidina ou do leucocianidol puros, compostos polifenóis isolados dos RWP, é de 8,91µg/mL e 1,77µg/mL, respectivamente.^17,18^ As substâncias identificadas em outros membros do gênero *Combretum *também são capazes de induzir ao EDR nas aortas de ratos. No entato, as concentrações necessárias são mais altas do que as de ECL; por exemplo, 5 a 80µg/mL (para o glicosídeo do ácido mólico isolado da folha de *C. molle*) e uma EC_50_ de 3,9µg/mL e 9,5µg/mL para o extrato metanólico das folhas de *C. celastroides* e das raízes de *C. racemosum*, respectivamente.^3,4^ Em contraste, na aorta de rato, 1,5µg/mL do ECL foi capaz de causar um relaxamento completo das contrações induzidas por PE. Os compostos vasodilatadores presentes no ECL provavelmente não estão relacionados àqueles já descritos em outros membros do gênero *Combretum*.

As células endoteliais podem constituir o principal sítio de ação dos compostos bioativos presentes no ECL. Essa sugestão se baseia na observação de que é necessário um endotélio intacto para causar o relaxamento em todas as artérias examinadas. Por outro lado, o ECL pode atuar nas células lisas vasculares, aumentando sua reatividade aos fatores de relaxamento produzidos basalmente pelo endotélio, como os inibidores da fosfodiesterase, mas os relaxamentos causados por esses inibidores ainda estão presentes nos anéis sem endotélio da aorta torácica de rato ou coelho.^19,20^ Na verdade, tal mecanismo foi proposto recentemente para o efeito relaxante de uma fração diclorometano de *Anogeissus leiocarpus*, um combretáceo.^21^

Considerando que a BK não tem qualquer efeito sobre os anéis da aorta torácica de coelho e de rato^6^ e que a histamina relaxa a aorta torácica de rato, mas não a de coelho,^22^ juntamente do fato de, nas artérias coronárias de porco, a ACh causar contração, sugere-se que os mecanismos envolvidos nessa ação são diferentes daqueles envolvidos na ação de agonistas de receptores que causam EDR. Além disso, as características cinéticas do relaxamento induzido por ECL, como um início mais lento e um maior tempo até atingir a estabilidade, distinguem claramente seu efeito daquele causado pela ACh, BK e histamina. Ademais, o fato de que o efeito permaneceu por pelo menos 4 horas após a remoção do extrato contrasta com o relaxamento temporário causado pela ACh ou BK, cujos efeitos já não são evidentes após 0,5 a 1 hora de sua remoção do banho.

Já que em algumas artérias (a artéria mesentérica superior e as artérias carótidas comuns de coelho, bem como a artéria coronária de porco e a aorta torácica de cobaias) o ECL é capaz de causar EDR na presença de inibidores de NOS, sugerimos que seu efeito está aparentemente relacionado à ativação das células endoteliais, para produzir qualquer fator de relaxamento que esteja sendo predominantemente produzido. Foi amplamente descrito que as células endoteliais são heterogêneas em termos de resposta a agonistas e dos fatores de relaxamento que são produzidos; por exemplo, as células endoteliais de pequenas artérias e arteríolas produzem principalmente EDHF, enquanto as células endoteliais da aorta torácica produzem quase que exclusivamente NO.^23^

Nossos resultados mostrando que uma grande parte de EDR induzidos por ECL que requerem a presença de Ca^2^ extracelular indicam que a ação do ECL está relacionada à ativação do influxo de Ca^2^ em células endoteliais. Apesar das vias que medeiam o influxo de Ca^2^ nas células endoteliais não terem sido inteiramente elucidadas, o fato de o RR ter sido capaz de reverter o relaxamento induzido por ECL sugere que um ou mais dos vários canais permeáveis de Ca^2^ sensíveis ao RR^24^ são ativados diretamente ou indiretamente pelos compostos bioativos presentes no extrato.

Existem evidências para sugerir que os compostos polifenóis podem constituir os princípios ativos responsáveis pelo efeito do ECL. Primeiramente, substâncias com reatividade química semelhante à dos polifenóis estão presentes no ECL em quantidades significativas (aproximadamente 40%m/m). Em segundo lugar, demonstrou-se que polifenóis derivados de plantas causam EDR em vários segmentos arteriais.^25^ Em terceiro lugar, estudos anteriores mostraram a presença de uma variedade de compostos, incluindo os polifenóis, em extratos etanólicos de flores, folhas, raízes, frutas e casca de *C. leprosum*, tais como flavonoides^12,26,27^, triterpenos,^26-28^ mono e oligossacarídeos, ácidos graxos, esteróis,^12,27,28^ e estilbenos.^29^

Finalmente, a descrição desse efeito vasorrelaxante de longa duração do causado pelo ECL pode ser clinicamente relevante, já que pode levar ao desenvolvimento de novas abordagens terapêuticas potenciais para condições patológicas nas quais o fluxo sanguíneo esteja reduzido por causa do tônus vascular aumentado, tal como a hipertensão e as doenças vasoespásticas. Mais experimentos são necessários para estabelecer o possível efeito terapêutico do ECL. Por exemplo, precisamos responder às seguintes perguntas: quais são os efeitos do ECL sobre a circulação sanguínea em animais intactos? Quais são os compostos ativos presentes no extrato? Quais são os mecanismos por meio dos quais o ECL promove a síntese e a liberação de fatores endoteliais?

## CONCLUSÃO

Os presentes achados mostraram, pela primeira vez, que uma suspensão aquosa de um extrato hidroalcoólico liofilizado de *Combretum leprosum* causa relaxamento de anéis arteriais isolados de diferentes espécies animais. Esse relaxamento foi completamente dependente de um endotélio funcional e parece ser mediado pela liberação de fatores relaxantes derivados do endotélio, tais como óxido nítrico na aorta torácica de rato ou de coelho, ou por óxido nítrico e outros fatores não derivados da atividade de óxido nítrico sintetase ou ciclo-oxigenase na aorta abdominal de coelho e nas artérias coronárias de porco. Finalmente, como o extrato de *Combretum leprosum* exige um influxo de Ca^2^ por meio de vias sensíveis ao vermelho de rutênio para causar o relaxamento, para a elucidação de seu mecanismo de ação, isso pode revelar uma nova abordagem para ativar células endoteliais a produzirem e liberarem fatores de relaxamento.
